# Daily breath-based mindfulness exercises in a randomized controlled trial improve primary school children’s performance in arithmetic

**DOI:** 10.1038/s41598-023-49354-0

**Published:** 2023-12-13

**Authors:** Katharina Voltmer, Finja Hondrich, Maria von Salisch

**Affiliations:** Institute for Sustainability Education and Psychology, Leuphana University Luneburg, Universitätsallee 1, 21335 Lueneburg, Germany

**Keywords:** Neuroscience, Psychology

## Abstract

Mindfulness-based interventions (MBIs) have been shown to improve children’s academic achievements. Because MBIs include different exercises (possibly with differential effects), the teacher-led Breathing Break Intervention (BBI) was developed which focuses exclusively on breathing exercises and body awareness. The short daily breathing practices of BBI were evaluated in terms of their effects on children’s performance in mathematics. In a randomized controlled trial, *N* = 140 third and fourth graders (49% female) either received BBI (IG, *n* = 81) or participated in an active control group (ACG, *n* = 59). Students took a standardized arithmetic test and teachers rated their mathematics performance before (T1) and after (T2) the nine weeks of BBI, and in a follow-up five months later (T3). A mixed multilevel model with a quadratic term of time indicated a significant interaction effect between group and time on the arithmetic test after controlling for working memory updating and parental educational attainment. IG children did not show a steeper linear increase but differed significantly from ACG children in their trajectory of arithmetic performance. At T3, IG children outperformed ACG children. A multilevel ordinal logistic regression of teachers’ ratings of students’ mathematics performance revealed no significant differences between IG and ACG. Results suggest that daily breathing exercises in primary school classrooms contribute to enhancing children’s performance in arithmetic.

Preregistration: The study was preregistered at aspredicted.org (#44925).

## Introduction

The COVID-19 pandemic at the beginning of 2020 and the resulting school closures have had a negative impact on children’s mathematics performance worldwide. Studies conducted in Germany^1^, the US^2^, Switzerland^3^, Belgium^4^, and the Netherlands^5^ have uniformly demonstrated a decline in primary school children’s mathematics performance during the first year of the pandemic. These findings are alarming because mathematics performance has repeatedly been related to future academic and occupational success^6,7^.

In recent years, mindfulness has received increased attention in education because of its positive effects not only on the well-being^8^ and school adjustment^9^ of young people but also on their achievements. A recent meta-analysis over 46 mindfulness-based intervention (MBI) studies with randomized controlled (RCT) designs demonstrated that MBIs contributed to a better school adjustment in samples from preschool to college (Hedges’ *g* = 0.19, *p* < 0.001). It also showed a small post-intervention effect over the 9 MBIs on academic performance (Hedges’ *g* = 0.19, *p* < 0.05)^9^. Another meta-analysis over 29 MBIs with quasi-experimental designs established a medium effect of MBIs on academic performance (Hedges’ *g* = 0.31, *p* < 0.001) in all grades and a small effect in grades 1 to 6 (Hedges’ *g* = 0.22, *p* < 0.05^10^). Other meta-analyses and systematic reviews have corroborated that MBIs in schools tend to enhance cognitive^11–13^, emotional^11,13,14^, motivational^12^, and social processes^12,13^ in children and adolescents. Each of these processes has been proposed as a pathway which contributes to children’s academic performance^10^. Examining the impact of an MBI on mathematics performance in third and fourth graders will provide evidence of how to improve their mathematical performance, facilitate their everyday life, and enhance their chances of future success.

### Mindfulness and mathematics performance

Mathematics encompasses the curricular sub-areas of arithmetic, geometry, quantities, and problem-solving^15^. The current study focuses on children’s achievements in mathematics in general and in the subfield of arithmetic, which addresses the properties and operations of numbers. Arithmetic in primary school involves the four basic operations of addition, subtraction, multiplication, and division of whole numbers.

Mindfulness originates from an ancient Buddhist practice of the Vipassana and the Zen tradition^16^. It revolves about being aware of the present moment and the associated contents of consciousness (i.e., bodily sensations, perceptions, thoughts, emotions). John Kabat-Zinn defined mindfulness as “the awareness that arises through paying attention, on purpose, in the present moment, and non-judgmentally”^16^. Kabat-Zinn developed the Mindfulness Based Stress Reduction (MBSR) program which has been shown to decrease the functional activity of the amygdala, to improve its functional connectivity with the prefrontal cortex and the hippocampus^16^, and to bring about structural changes in the amygdala^17^. MBSR-based MBIs are increasingly implemented and support the impact of MBIs on top-down and bottom-up self-regulation in preadolescent children^18^.

MBIs have been shown to affect children’s performance in mathematics. In a quasi-experimental study, sixth graders in the intervention group (IG) participated in an audio based MBI for 18 weeks. At the end of the school year, the IG performed better in a standardized test of mathematics than their agemates in the control group^19^. A RCT evaluating a MBI with fourth and fifth graders demonstrated that mathematics grades improved as a trend when the IG was compared to an active control group (ACG)^20^. Zuo and Wang^21^ confirmed that an audio-based MBI contributed to the improvement of eighth graders’ math performance in high-stakes testing in China. Correlational studies indicated moderate positive associations between self-reported mindfulness and mathematics performance in middle school students^22^. Another quasi-experimental study concluded that participation in an MBI for eight weeks contributed to the explanation of third and fourth graders’ quarterly grades in reading and science, but not in mathematics^23^. Nevertheless, neuropsychological evidence suggests that practicing mindfulness may improve mathematics performance: A fMRI study underlined that better arithmetic competence in children was associated with increased activity in the intraparietal sulcus^24^. Studies with adults show that meditations with mindfulness components tend to encourage increased activity in this and closely related regions of the brain^25,26^.

### The current study

Because MBIs in school include a variety of practices or components of mindfulness with differential effects^27^, this study concentrates on children’s experience of breathing and observing sensory experiences^9^ which are basic techniques of “body-centered meditation”^28^ or of the mindfulness practice group 1 which centers on “cultivating attention to the present moment somatic and sensory experiences”^29^. Because these exercises can be performed with minimal verbal elaboration, they are well-suited for primary school children who tend to struggle with the metacognitions which are needed for verbally-based mindfulness interventions that rely on observation and verbalization of their thoughts and feelings. In our Breathing Break Intervention (BBI), teachers encouraged their students to participate in 3- to 5-min breath-based mindfulness exercises up to 3 times per school day. Further details on BBI can be found in the Method section.

Few studies have examined the relations between mindfulness and mathematics performance in youth. Many of them were limited by methodological concerns, such as lack of directions of effects in correlations with trait mindfulness, the use of teachers’ grades to measure mathematics performance, and a focus on short-term effects. Additionally, most of the participants were beyond primary school age. The current study aims to fill these gaps by experimentally investigating the effect of BBI on primary school children’s mathematics performance in a RCT with an Active Control Group (ACG) and three measurement points, i.e., a pretest before (T1), a posttest after the BBI (T2), and a follow-up five months later (T3). Both subjective assessments of children’s performance by their mathematics teachers and an objective achievement test were used.

Children’s Working Memory Updating (WMU) and their families’ socioeconomic status (SES) were controlled because these variables are known to influence children’s mathematics performance^30,31^. Children’s WMU is a component part of their executive functions. Having a large “storage space” for WMU makes it easier for children to manipulate incoming numbers, such as applying multiplication rules. WMU was controlled because interindividual differences in WMU tend to influence mathematics performance and may obscure the effects of the BBI. Because parents of higher SES tend to provide their children with financial support, a more stimulating home environment for learning, and with social support and instructions about the behaviors that are valued at school, their children’s achievements in mathematics tend to be higher. This has been demonstrated time and again in the PISA studies^30^. In order to rule out that effects of the BBI on children’s performance in mathematics were concealed by children’s families’ SES, this variable was controlled.

The present research thus aims to answer the following question: *How does the BBI affect primary school children’s performance in arithmetic and teacher ratings of general mathematics?* Two hypotheses are proposed: (1) We expect a greater improvement in arithmetic performance after the BBI for children in the IG than the ACG when controlling for their parents’ educational attainment (as a proxy for SES) and WMU and (2) we expect a greater improvement in teacher ratings of children’s general mathematical performance for children in the IG than the ACG when controlling for parents’ educational attainment and WMU. This will be demonstrated by significant interactions between group (i.e., IG vs. ACG) and time in multilevel regression analyses.

## Method

### Participants

**Figure 1:**
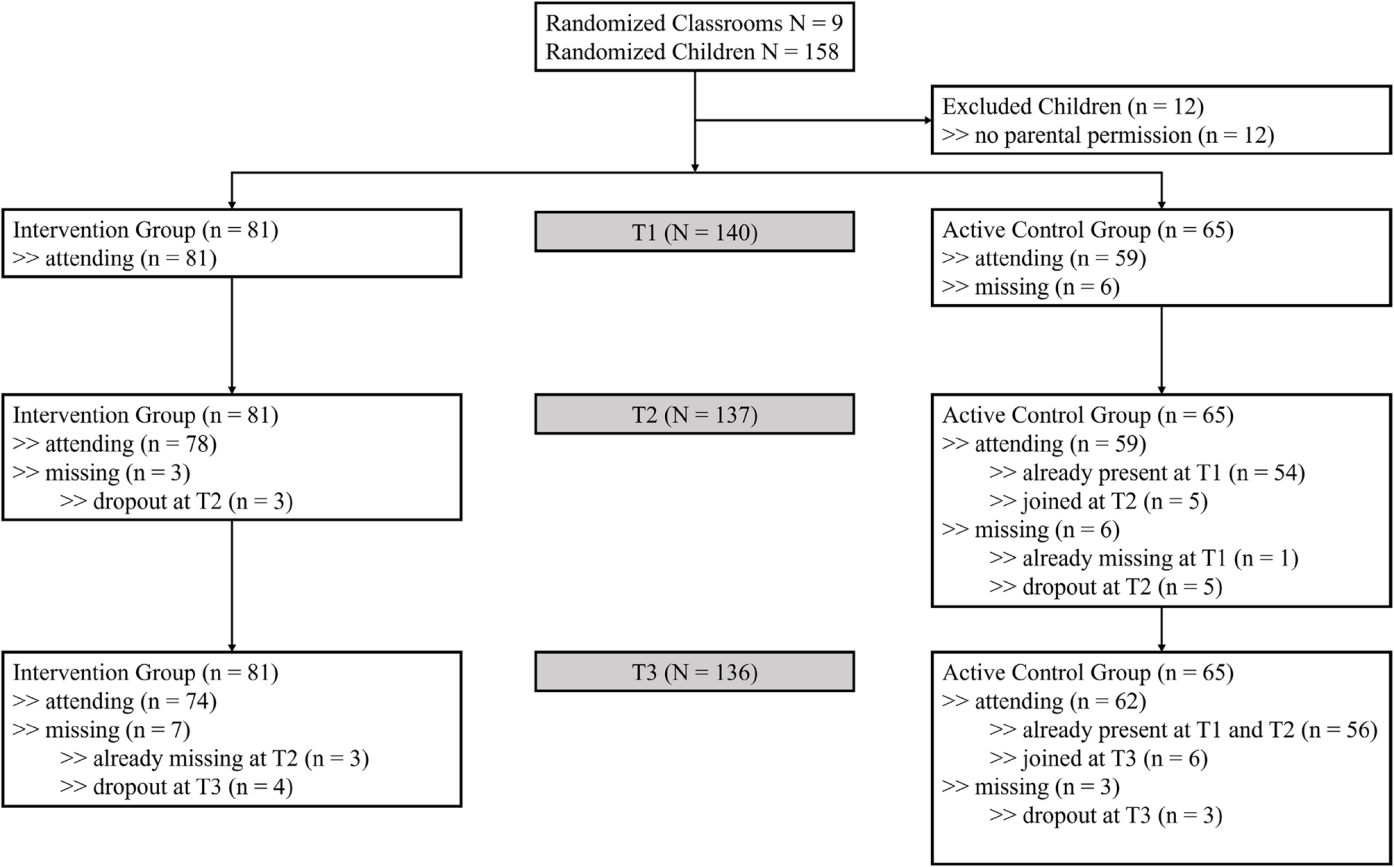
The recruitment flow of the sample for the present study. At each measurement point few children drop out, mainly due to illness or refusal. However, at T2 and T3 some children joined the sample after having missed the previous timepoint(s).

Written invitations and information flyers were sent to 26 elementary schools in and around a medium-sized city in Lower Saxony, Germany. In the end, nine classroom teachers from six schools participated in the study. To prevent the sharing of intervention content between the teachers (or the children) from the two groups, all classes in one school were either assigned to the IG or the ACG. Schools were randomly assigned to the IG or the ACG. This resulted in three schools with five classes of the third grade for the IG, whereas three schools with two third grade classes and two fourth grade classes made up the ACG. At T1 data was collected from *N* = 140 children. Among them, *n* = 81 were in the IG and *n* = 59 were in the ACG. Figure 1 shows that a few children had missing data at one or two measurement points. As a result, the sample sizes for the T1, T2, and T3 data collection were *N* = 140, *N* = 137, and *N* = 136, respectively. Missing data and dropouts were primarily due to illness, relocation to another city, or refusal to participate. Participation rates for the T2 and T3 data collections were 98% and 97% of the T1 sample.

The sociodemographic characteristics of the sample at T1 can be found in Table 1. Children in the ACG (*M* = 8.76 years, *SD* = 0.80) were significantly older than in the IG (*M* = 8.31 years, *SD* = 0.56, t(138) = 3.95, *p* < 0.001) because half of the ACG children were in fourth grade classrooms, whereas all IG children attended third grade classrooms. Neither the proportion of boys and girls nor parents’ educational attainment nor family migration nor the proportion of children from bilingual families nor the proportion of children with special educational needs (Learning, *n* = 4; Physical Development, *n* = 1; Emotional Development, *n* = 3, Chronic Illness, *n* = 1 with one child having two different needs) differed between the ACG and the IG. ACG children reported more often at T1 that they had engaged in breathing exercises on their own than IG children.

**Table 1:** The sociodemographic background of the children in the IG and the ACG at T1 with percentages in parentheses.

	IG *N* (%)	ACG *N* (%)	Total *N* (%)	Test statistic^a^
Gender				*X*^*2*^(1) = 1.73, *p* = 0.188
Female	35 (43)	33 (56)	68 (49)	
Male	46 (57)	26 (44)	72 (51)	
Parents’ educational attainment				*Χ*^2^(1) = 1.32, *p* = 0.251
No vocational qualification	14 (17)	16 (27)	30 (21)	
Vocational qualification	63 (78)	41 (70)	104 (74)	
Missing^b^	4 (5)	2 (3)	6 (4)	
Immigrant family				*Χ*^*2*^(1) = 1.76, *p* = 0.185
No	65 (80)	42 (71)	107 (76)	
Yes	13 (16)	16 (27)	29 (21)	
Missing^b^	3 (4)	1 (2)	4 (3)	
Bilingualism				*Χ*^*2*^(1) = 1.98, *p* = 0.160
No	69 (85)	43 (74)	112 (81)	
Yes	12 (15)	15 (26)	27 (19)	
Special educational needs				Fisher’s exact test *p* = 0.721
No	77 (95)	55 (93)	132 (94)	
Yes	4 (5)	4 (7)	8 (6)	
Prior experience with breathing exercises				Mantel–Haenszel *Χ*^*2*^(1) = 4.21, *p* = 0.040
Never	65 (80)	36 (61)	101 (72)	
Once a month	5 (6)	8 (14)	13 (9)	
Once a week	5 (6)	9 (15)	14 (10)	
2–4 times a week	3 (4)	5 (9)	8 (6)	
5–7 times a week	2 (3)	1 (2)	3 (2)	
Missing^b^	1 (1)	0 (0)	1 (1)	

Compares the IG and the ACG in terms of gender, parents’ education, immigrant family, bilingualism, special educational needs, and prior experience and finds no statistical differences between the two groups. There was only one significant difference: ACG children tended to have more experience with breathing exercises than IG children.

*IG* intervention group, *ACG* active control group, *N* sample size, *SD* standard deviation.

^a^Unless otherwise stated the test is a Pearson’s Chi-square test.

^b^Missing values were not included as a group in group comparisons.

### Procedure

After receiving approval from the Ethics Review Board (Beirat für Ethikfragen in der Forschung) of the authors’ university on May 6, 2020, that all research was performed in accordance with relevant guidelines and regulations, T1 data collection took place in September 2020. T2 data were collected in December 2020, just a few days before the second COVID-19 pandemic-related lockdown in Germany. The T3 assessment took place in May 2021. Because of the pandemic, children’s attendance at school between T2 and T3 was limited. In order to limit infections, most classes were split up into two “learning groups”, with each group attending school for two or three days per week. Data were collected with the help of tablet computers, with a test manager in front of the class who read the items and instructions. Children could read along silently. The working memory updating task was taken in small groups. Trained undergraduate research assistants supported the children who encountered technical difficulties with the tablets. More details can be found in von Salisch and Voltmer^15^.

Data collection took three to four teaching hours at each measurement point, with the usual breaks in between. Informed consent was obtained from all child subjects and their legal guardian(s). Their participation was voluntary. At each measurement point, all children received a small gift after finishing the data collection. Teachers completed rating scales for each child of their class and paper–pencil questionnaires about themselves. Parents completed a brief questionnaire regarding their family situation, the languages spoken at home, and their educational and professional backgrounds. At each measurement point, children took the objective test of arithmetic in their regular math lesson under the supervision of their math teacher.

In June 2020, the nine teachers of the IG received the manual of the newly developed BBI curriculum and participated in a short mindfulness training. During the 15 h of the training, they were introduced to the concept of mindfulness, practiced key mindfulness exercises, and received guidance on leading the breathing exercises for the children in their classes. Teachers' implementation of breathing exercises with the children was supervised by a certified MBSR teacher with extensive experience in teaching mindfulness in primary schools. The BBI was conducted between T1 and T2 for approximately nine weeks in all IG classrooms. Teachers invited their students to perform one of the 15 activating or calming breathing exercises or body awareness exercises of the BBI up to three times per day, every school day. There was no pre-determined order or sequence. Exercises had attractive names. Teachers were free to choose the exercise which fit best the current classroom situation. A short description of the learning objectives for each group of exercises is provided in von Salisch and Voltmer^15^. In order to limit infections, schools were closed for about two months after T2 and children received distance education. In the following three months, most classrooms were split into two “learning groups”, so that children spent much less time in school. Because there was a lot of pressure to “catch up” on lessons, Breathing Breaks were rarely performed in school.

During the intervention period, the ACG teachers were asked to guide the children to color a mandala for three to five minutes, up to three times each school day. Mandalas were chosen because it is a quiet and easy activity that requires no training. Because the ACG teachers did not receive any training on how to conduct the coloring, it was not considered a mindful activity. Teachers were aware of the fact that their classes were in the ACG condition.

### Measures

#### Arithmetic performance

The German Lernverlaufsdiagnostik-Mathematik für zweite bis vierte Klassen (LVD-M 2–4) [Curriculum-based Measurement-Mathematics for Second to Fourth Grades] by Strathmann and Klauer^32^ was used to assess level and development of children’s elementary mathematics calculation skills. The test consists of a total of 24 items—six for each arithmetic operation (addition, subtraction, multiplication, division). In LVD-M 2–4, each child receives an individualized set of items which is randomly generated by a software which comes with the test. In second grade, mental arithmetic involving addition, subtraction, multiplication, and division within a number range up to 100 is assessed. In third grade, mental arithmetic in all four mathematics operations in addition to written arithmetic tasks involving addition and subtraction within a number range up to 1000 are tested. In fourth grade, students are asked to solve mental arithmetic problems in addition and subtraction, as well as written arithmetic in all four mathematics operations up to 10,000. Correct answers were summed up. The minimum score was 0 and the maximum score was 24. German norm data are available so that the raw scores at each measurement point could be translated into T-scores. Because children’s raw scores were compared to those of their “grade mates” from the norming sample, attending a third or fourth grade classroom was controlled for. The T-norm always has a mean of 50 and a standard deviation of 10 and requires a normal distribution of scores. As a result, values below 40 are below average and values above 60 are above average.

Because of the first Covid-19 related lockdown and associated delays in learning in the preceding school year, teachers expressed concern that many of their students were not able to meet grade-level expectations and would be frustrated by a difficult test. Therefore, at T1 the test versions of the previous grade were used, i.e., third graders took the test for second graders and fourth graders that for third graders. T-scores from the end of second grade were used to evaluate third graders’ performance at T1, whereas T-scores from the end of third grade were utilized for fourth graders’ performance at T1. At T2 and T3, however, grade-appropriate arithmetic tests and T-scores were employed. At T2 (December) T-scores for the middle of the school year were used. T-values for the middle of the school year were also chosen for T3 (May) because the usual growth in learning could not be assumed due to the second Covid-19 related lockdown. Criterion validity of the LVD-M 2–4 was established by high correlations with another standardized mathematics test (*r* = 0.53 to *r* = 0.80) in the norming sample. LVD-M 2–4 also correlated negatively with the math grade (*r* = − 0.54 to *r* = − 0.77) because higher numbers correspond to lower grades in the German school system^32^. The test was administered by the mathematic teachers of the classrooms. Because the test was not conducted on the same day as the rest of the data collection, different numbers of children were present. Therefore, the sample sizes of the math test differ from those of the rest of the data collection.

#### Teacher ratings of mathematics

At all three measurement points, teachers rated children’s overall mathematics performance with one item (“How do you rate the student’s level of achievement in mathematics?”) on a scale ranging from 1 to 5 (strongly below average [1], below average [2], average [3], above average [4], strongly above average [5]).

#### Working memory updating (WMU)

Children’s WMU was measured through the backward digit span of the app version of the Eichstätter Messung des Arbeitsgedächtnisses (EI-MAG; Eichstatt Measurement of Working Memory^33^). In this objective test, the digits 1 to 9 were presented via headphones in intervals of 1.5 s. Afterwards, a 3 × 3 matrix of Arabic numerals appeared on the tablet screen with the digits 1 to 9. Children were instructed to enter the digits of the previously heard sequence in reverse order into the keyboard of the tablet. The number of correct answers (accuracy) was tallied. In the adaptively designed EI-MAG, the number of digits to be remembered increased which raised the level of difficulty. Children were asked to reproduce two out of the three series of the same length in each block correctly before they could proceed to the next level. In the first block, two digits were to be remembered. In the most difficult block, a span of eight digits had to be reproduced. The test ended when children had made three mistakes within one block. The EI-MAG started with a practice phase. All children who achieved zero correct answers during the practice phase were excluded from the analyses because it was assumed that they had not understood the instruction.

#### Children’s acceptance

IG children’s acceptance of the Breathing Breaks was obtained at T3 by a short “Consumer satisfaction questionnaire”. In this questionnaire, children were asked to indicate (among other things) how often they wanted to continue the Breathing Breaks in the classroom. Children were also asked whether they had shown the breathing exercises to family members and friends and how frequently they had performed the Breathing Breaks “on their own”, i.e., outside of class, since T2 about five months earlier. Because the school closures and distance education occurred in the months between T2 and T3, children were also asked “Did the Breathing Breaks help you in coping with the strains (associated with remote education)?”.

#### Socioeconomic status

Children’s socioeconomic status (SES) was operationalized by their parents’ highest educational attainment which was assessed on a scale of 1–7 (no degree [1], secondary school degree (lower track) [2], secondary school degree (middle track) [3], high school diploma [4], apprenticeship [5], technical college [6], university degree [7]) which they had self-reported. Subsequently, a dichotomous scale was derived, which categorized the participants into two groups: those without a vocational qualification [1] and those with a vocational qualification [2].

#### Teachers’ implementation calendar

For each school day between T1 and T2, IG teachers wrote down the exercises they had performed with their class in an implementation calendar. Because the exact number of intervention days varied between classrooms, the number of maximally possible Breathing Breaks (3 * number of intervention days) varied between 148 and 164. When calculating the proportion of actually delivered Breathing Breaks (between 65 and 149) out of the maximally possible Breathing Breaks for each classroom, it was established that teachers led 41%, 48%, 77%, 84, and 91% of all possible Breathing Breaks in IG classrooms. The breathing exercises were not performed because of teachers’ illness, project days, or a COVID-19 quarantine of the whole classroom.

#### Teachers’ acceptance

Teachers were asked at T3 to report in retrospect how many Breathing Breaks they had led with their class since T2 about five months earlier, whether they benefited from the Breathing Breaks themselves, whether they noticed a change in their relationships with the children, whether they would like to lead the breathing exercises with future classrooms, and whether they considered the Breathing Breaks to be helpful. A quarter of the IG teachers reported at T3 that they had not led any Breathing Breaks between T2 and T3. Fifty percent reported that they had delivered Breathing Breaks approximately once a month and 25% reported performing them once a week or more often.

## Results

### Descriptive statistics

#### Correlations

Correlations for all study variables across all measurement points are presented in Table 2. By excluding the children who scored zero points in the WMU practice trial, the sample size for WMU decreased by 28 children at T1, and by 11 children at T2 and T3. Correlations between arithmetic test scores and teacher ratings of mathematics performance were moderate and those with WMU were small in magnitude. Correlations between arithmetic test scores and parents’ educational attainment were medium sized. Correlations between control variables and dependent variables were significant and varied from small to strong in magnitude. No significant correlations between gender and arithmetic test scores and teacher ratings in mathematics emerged. Therefore, gender will not be considered any further.

**Table 2:** Holm adjusted Spearman correlations for study variables for all measurement points.

	Gender	PEA	IF	T1 WMU	T2 WMU	T3 WMU	T1 AT	T2 AT	T3 AT	T1 TR	T2 TR
Gender											
PEA	− 0.15										
IF	0.03	− 0.51***									
T1 WMU	0.10	0.19	− 0.16								
T2 WMU	0.14	0.08	− 0.12	0.19							
T3 WMU	0.18	0.21	− 0.11	0.32*	0.15						
T1 AT	− 0.07	0.41***	− 0.24	0.22	0.34*	0.29					
T2 AT	− 0.16	0.36**	− 0.18	0.08	0.26	0.26	0.66***				
T3 AT	− 0.05	0.24	− 0.19	− 0.03	0.20	0.23	0.50***	0.60***			
T1 TR	− 0.14	0.12	− 0.02	0.11	0.19	0.06	0.33**	0.34**	0.29*		
T2 TR	− 0.21	0.28	− 0.13	0.23	0.34*	0.23	0.43***	0.52***	0.50***	0.49***	
T3 TR	− 0.20	0.34**	− 0.22	0.29	0.32*	0.26	0.50***	0.58***	0.60***	0.47***	0.88***

The correlations between the study variables gender, parents’ educational attainment (PAE), immigrant family (IF), working memory update (WMU), arithmetic test (AT), and teacher ratings of mathematics (TR). Correlations with WMU, AT, and TR are displayed at T1, T2, and T3. Since gender did not correlate with any other variable, it will not be considered any further. Because PAE correlated highly with growing up in an immigrant family (*r* = − 0.51, *p* < 0.001), only PEA will be considered in the following statistical analyses. Because PEA correlated with AT at T1 and T2 and with TR at T3, it will be included as a control variable. The other control variable is WMU which correlated with AT (at T1) and TR (at T2 and T3). Correlations between AT and TR were medium in size.

*PEA* parents’ educational attainment, *IF* immigrant family, *WMU* working memory updating, *AT* arithmetic test, *TR* teacher rating in mathematics.

*N* = 99–140. S

*p < 0.05; **p < 0.01; ***p < 0.001.

#### Arithmetic test performance

**Figure 2:**
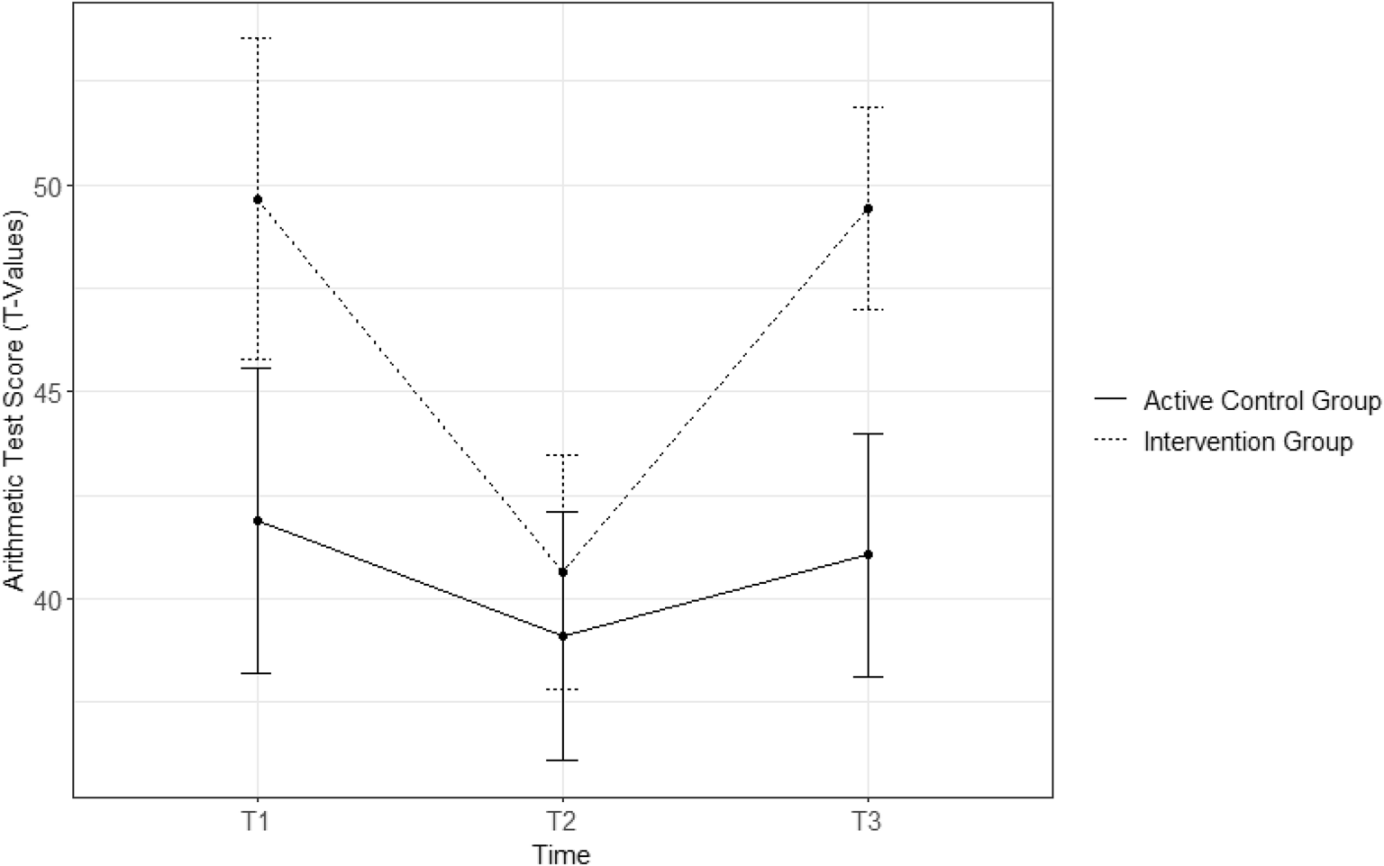
Means and 95%-confidence interval of arithmetic test scores (T-values) over time for the intervention group and the active control group. The graphic representation of the (grade-corrected) T-values of IG and ACG children’s scores on the arithmetic test and their confidence intervals at the three points of measurement. Visual inspection suggests that children in the IG outperformed children in the ACG at T1 and T3 because confidence intervals of the two groups do not overlap at these measurement points.

For the arithmetic test, at T1 data were available for *n* = 133, at T2 for *n* = 127, and at T3 for *n* = 136. When examining the trajectory of the arithmetic test scores in Fig. 2, it becomes evident that the IG started higher at T1 than the ACG. Subsequently, performance dropped. Both groups aligned in their performance at T2 and increased it at T3, but with a steeper slope in the IG than in the ACG. Comparisons of the test scores of both groups in Table 3 indicate that IG children surpassed ACG children in their arithmetic test performance at T3. Boys and girls did not differ in their test scores at T1 (*t*(131) = 0.54, *p* = 0.588).

**Table 3:** Descriptive statistics and comparison of arithmetic test scores (t-values) in the intervention group and active control group across all measurement points.

	IG			ACG			Test statistic
	*n*	*M*	*SD*	*n*	*M*	*SD*	
T1	78	49.65	17.51	55	41.87	13.94	*t*(131) = − 2.74, *p* = 0.007
T2	74	40.65	12.33	53	39.09	11.14	*t*(125) = − 0.73, *p* = 0.467
T3	78	49.44	10.97	58	41.07	11.40	*t*(134) = − 4.33, *p* < 0.001

The means and standard deviations of the arithmetic test scores of the children in the IG and the ACG across the three measurement points are compared with t-tests. The t-tests suggest that the IG outperformed the ACG both at T1 and T3.

*IG* intervention group, *ACG* active control group, *n* sample size, *M* mean, *SD* standard deviation.

#### Group differences in arithmetic test performance over time

For the longitudinal analysis of the arithmetic test scores, a multilevel mixed effects model with random slopes was calculated in R^34^ with the *nlme* package^35^. The arithmetic test scores at the three measurement points represented level one and were nested in the children at level two. The children were nested in their classrooms, which formed level three. In the analysis, 129 children in 9 classrooms led to 317 observations. Because visual inspection of the data revealed a U-shaped trajectory of the test scores (Fig. 2), a quadratic term of time was added to the model. The interaction between group (IG/ACG) and time was included as predictor of arithmetic test scores whereas WMU and parents’ educational attainment served as control variables.

As displayed in Table 4, neither the linear nor the quadratic main effect of time (time^2^) nor the main effect of group membership were significant predictors of children’s arithmetic test scores. Children with higher WMU scores and children with at least one parent with a vocational qualification scored higher on the arithmetic test independent of time. The interaction effect between the group and the quadratic term of time indicates that the trajectories of arithmetic test scores differed between the IG and the ACG while controlling for the control variables. Figure 2 shows that IG children initially showed a greater decrease in their performance between T1 and T2 and then a greater increase between T2 and T3 than the ACG children.

**Table 4:** Interaction effect between group membership and time on arithmetic test scores in a quadratic multi-level model.

	β	SE	df	t	*p*
Intercept	33.086	3.217	183	10.284	***
Time	− 5.666	3.879	183	− 1.461	
Time^2^	1.973	1.845	183	1.069	
Group (Intervention = 1)	6.537	3.087	7	2.117	
WMU	1.247	0.397	183	3.142	**
PEA (Vocational = 1)	7.297	2.355	119	3.098	**
Group * Time	− 14.442	4.924	183	− 2.933	**
Group * Time^2^	7.609	2.344	183	3.246	**

The results of a quadratic mixed effects model are displayed in Table 4. The t-tests suggest that WMU and parents’ educational attainment show a main effect of arithmetic test scores, but that neither the linear nor the quadratic representation of time affected these scores. Belonging to the IG or the ACG did not show a main effect on the test scores either. The interaction between group membership and the quadratic effect of time suggests that IG children outperformed ACG children in their arithmetic test scores over time.

*β* beta coefficient, *SE* standard error, *df* degrees of freedom, *t* t-value, *p* probability. *WMU* working memory updating, *PEA* parents’ educational attainment.

***p < 0.001; **p < 0.01; *p < 0.05.

The results of this quadratic mixed effects model provide valuable insights into the variability explained by the fixed and random effects. The conditional R-squared exhibited a substantial value of 0.567, indicating that approximately 57% of the total variance in the arithmetic test performance were accounted for by both the fixed and the random effects of the model. A considerable portion of the observed variance was explained therefore not only by the fixed influence of parents’ educational attainment and children’s WMU but also by the child-specific and classroom-specific variability that was captured by the random effects. The marginal R-squared exhibited a value of 0.168, reflecting that approximately 17% of the variance could be attributed solely to the fixed effects of parents’ educational attainment and children’s WMU. The marginal R-squared provides insight into the unique contribution of these predictors to children’s arithmetic performance, independent of the random effects. Altogether, these R-squared values highlight the strong performance of the overall model and the usefulness of combining both fixed and random effects in one model.

#### Post-hoc analyses of arithmetic performance

To ensure that these results were not due to the combined analysis of data from third and fourth grade students, the analyses were repeated without including the children from fourth grade. The results of the quadratic multi-level model remained essentially the same in this reduced sample.

In a linear regression model, the proportion of actually delivered Breathing Breaks out of the maximally possible Breathing Breaks for each classroom of the intervention group between T1 and T2 just missed having a significant effect on children’s arithmetic performance at T3 when controlling for parents’ educational background and children’s WMU scores (*t*(*81*) = 1.916, *p* = 0.61). The number of Breathing Breaks performed in each classroom was thus not related to children’s arithmetic performance.

#### Teacher ratings of mathematics

**Figure 3:**
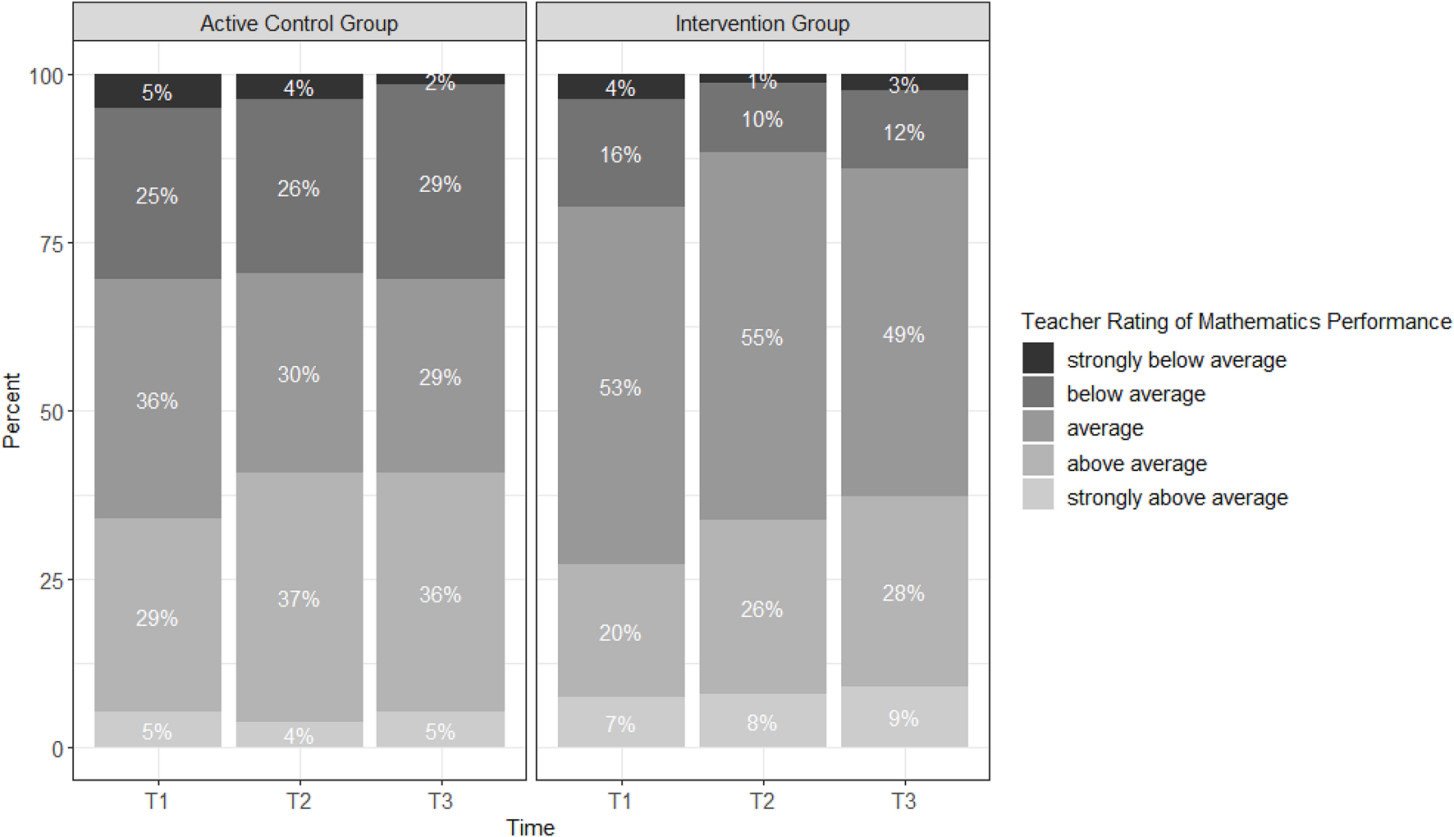
Teacher ratings of the mathematics performance of children in the active control group and the intervention group over three measurement points. The distribution of the percentages of teacher ratings indicates slight differences between the two groups.

Teachers provided ratings of mathematics performance at T1 for *n* = 140, at T2 for *n* = 131, and at T3 for *n* = 137 children. No gender differences in teacher ratings at T1 were found (Mantel–Haenszel *Χ*^2^(1) = 2.30, *p* = 0.129). The distribution of teacher ratings of children’s mathematics performance in both groups over time is displayed in Fig. 3. Teacher ratings did not differ significantly between the groups at T1 (Mantel–Haenszel *Χ*^2^(1) = 0.24, *p* = 0.628).

#### Group differences in teacher ratings in mathematics over time

Teacher ratings in mathematics were analyzed with a multi-level ordinal logistic regression model with the package *ordinal*^36^ because the outcome variable was an ordinal scale. This model had the same three level structure as the mixed effects model above. The data of 130 children in 9 classrooms led to 330 observations. Again, the interaction between time and group (IG/ACG) was included as predictor of mathematics ratings with WMU and parents’ educational attainment as control variables. Table 5 indicates that neither the main effect of time nor the interaction effect of time and group contributed to the explanation of teachers’ math ratings. Thus, independent from being in the IG or ACG, it was not more likely for children to be rated higher or lower in their math performance by their teachers over time. Again, WMU and the parents’ educational attainment were significant predictors, which had a positive impact on teachers’ ratings independent of time.

**Table 5:** Interaction effect between group membership and time on teacher ratings in mathematics in a multi-level ordinal logistic regression analysis.

	β	SE	z
Time	0.436	0.240	1.815
Group (Intervention = 1)	0.823	0.843	0.977
WMU	0.234	0.098	2.375*
PEA (Vocational = 1)	1.821	0.697	2.613**
Group * Time	− 0.069	0.311	− 0.223

The results of a multi-level ordinal logistic regression analysis which suggests that WMU and parents’ educational attainment had a main effect on teachers’ ratings of children’ performance in mathematics. The interaction effect between group membership and time was not significant.

*β* beta coefficient, *SE* standard error, *df* degrees of freedom *t* t-value. *WMU* working memory updating, *PEA* parents’ educational attainment.

***p < 0.001; **p < 0.01; *p < 0.05.

Whereas R-squared is a commonly used measure for assessing model fit in ordinary linear regression, it is not directly applicable to the current modeling approach. Mixed-effects ordinal regression involves both fixed and random effects. This creates a challenge when it comes to obtaining a straightforward R-squared value that represents the proportion of variance explained. Instead, we relied on alternative indices to assess the model's fit. The Akaike Information Criterion (AIC) and Bayesian Information Criterion (BIC) of the current model was compared to one without the interaction effect between time and group in terms of their relative fit. The likelihood ratio test indicated a significant difference between the model without the interaction effect (AIC = 740.6, logLik = − 361.30) and model with the interaction effect (AIC = 738.1, logLik = − 358.05), χ^2^(2) = 6.5048, *p* = 0.039. This indicates that the inclusion of the interaction term in the model significantly improved the model fit. Additionally, Nakagawa's R^2^ (pseudo R^2^) was computed which indicates the variance explained by the model. The conditional pseudo R^2^ of 0.721 revealed that approximately 72% of the total variance in children’s teacher-rated mathematic performance was explained by both the fixed and the random effects of the model, which comprise the predictors and the variability between the groups, respectively. This suggests that a substantial amount of variance was accounted for by the model. The marginal pseudo R^2^ of 0.086 represented the proportion of variance in the teacher-rated mathematic performance that was explained by the fixed effects alone (i.e., without considering the random effects). While the marginal R^2^ was comparatively low at 0.086, it still indicated the modest amount of variance explained by the fixed predictors in the model.

## Discussion

The present study examined the impact of the Breathing Break Intervention (BBI) on primary school children’s mathematics performance. IG children did not show a steeper linear increase but differed significantly from ACG children in their overall trajectory of arithmetic performance. In particular, a significant interaction effect between membership in the IG or the ACG and the quadratic term of time for the arithmetic test scores was established. Visual inspection of the test results in Fig. 2 suggests that the decrease in performance between T1 and T2 was stronger in the IG than in the ACG, but that the IG showed a steeper learning curve between T2 and T3 than the ACG. Table 3 shows that IG children outperformed ACG children in arithmetic at T3. Under normal circumstances curriculum-based arithmetic tests show a linear increase over a school year^32^. Because of the loss of class time due to the first lockdown of the COVID-19 pandemic, however, teachers were afraid that the children had not yet mastered the content of the previous grade. This is why the arithmetic test of the previous grade was used at T1 in September 2020. A T1 score of 50 indicated that IG children had reached the educational objectives of the previous grade on average, whereas the ACG children scored below average (~ 42 points). The T2 data do not show a real decline in mathematical performance, because the T1 test was “too easy” for all children. When using the grade-appropriate test at T2, both groups scored below average in relation to the mid-year norm data. These norms were a bit too difficult, given that T2 took place at the end of November and the beginning of December, i.e., one to two months before the middle of the school year at the end of January. The low scores at T2 can also be explained by the many measures designed to fend off COVID-19 infections in the preceding months, like airing classrooms, washing hands, wearing masks, taking COVID-19 tests, and fewer lessons because of quarantine measures. The steeper linear increase in arithmetic performance of the IG (see Fig. 2 and Table 3) can be observed when comparing their scores to those of the ACG at T2 to T3, which both used the same grade-appropriate test and the same mid-year norm data. All in all, the significant interaction effect between group membership and the quadratic term of time confirmed the first hypothesis insofar as children in the IG showed a stronger improvement in their arithmetic performance between T2 and T3 than children in the ACG when controlling for their parents’ educational attainment and WMU. This finding is in line with the results by Stager^19^ and the RCT by Schonert-Reichl et al.^20^.

Engaging in practices of mindfulness on a regular basis is likely to improve children’s mathematics performance through (1) cognitive, (2) emotional, (3) motivational, and (4) social processes^10^. *Cognitive processes*, such as Executive Functions (EFs) include working memory updating^37^ which seems to be pivotal for solving mathematical problems and mental arithmetic tasks^30^. Meta-analytic evidence indicates that MBIs tend to strengthen children’s EFs^11,13^ by inviting them to tame their wandering mind and to focus their attention on the present moment. Neuropsychological evidence supports that MBIs tend to foster volitional top-down self-regulation (through EFs)^18^. With a better command of their EFs, children are better able to solve mathematical problems.

In the Debilitating Anxiety Model^38,39^ e*motional processes*, such as negative thoughts (which are typical of math anxiety) tend to interfere with children’s working memory operations^40^ and contribute to a lower performance in mathematics^41,42^. MBIs can teach children to become aware and let go of their anxiety without further rumination. Qualitative interviews after a MBI on mathematics anxiety in China confirmed that the participants observed fewer physiological manifestations of anxiety, less test-irrelevant worry, and fewer obstacles to solving mathematical problems while at the same time gaining more math self-efficacy^21^. Meta-analyses agree that MBIs contributed to improving children’s socioemotional functioning, which included anxiety, stress, emotion regulation, and internalizing behaviors^11,13,14^. Neuropsychological evidence corroborated the impact of MBIs on bottom-up self-regulation^18,43^ including structural changes in adolescents’ amygdala^44^ and amygdala reactivity^45^. When MBIs help youth to reduce interfering negative thoughts (about math), they can use all their cognitive resources to solve the mathematical problems, which enhances their performance.

Enhanced EFs and less interference of negative thoughts are prerequisites for the *motivational process* of self-regulation^46^ which seems to bolster mathematics performance as well^47^. Another motivational predictor of mathematics performance is the *academic self-concept* which is the assessment of one's own academic abilities. Participation in MBIs seems to generate a more benign self-appraisal^12^ and to promote the maturation of brain regions involved in cognitive control and self-regulation^48^. MBIs seem to have a positive impact on self-regulation and academic self-concept which are positively related to mathematics performance.

Social processes, such as prosocial behavior and a supportive classroom climate, can also enhance children’s academic performance^49^ because they promote cooperation^50^. Positive reinforcements from classmates tended to strengthen children’s academic self-concept^49^. Meta-analyses underline that MBIs tend to foster prosocial behavior^13^ and the social climate in the classroom^51^. These effects were confirmed in the evaluation study of the BBI^15^. MBIs thus tend to foster prosocial behavior and classroom climate which are likely to facilitate children’s performance in mathematics.

The present study is in line with the meta-analyses^9,10^ that confirmed a small to medium effect of MBIs on students’ academic achievements in (randomized) controlled study designs. That many effects were small may be explained by the large number of studies which only examined short-term effects^10^. Because many third and fourth graders are still limited in their inhibition^52^ they may have needed more practice time to develop their inhibition before they could fully embrace mindfulness^18^.

In Verhaeghen’s meta-analysis^10^ more hours of practice were associated with stronger effects on academic performance when mindfulness was practiced at home. This is rarely observed but speaks for children’s motivation^53^. The effects of BBI on children’s arithmetic scores can be explained by a prolonged home practice for many children. When asked at T3, 40% of the IG children pointed out that they had continued the Breathing Breaks during the months of remote education between T2 and T3. 18% had practiced “once a week” or more often at home. 45% of the children indicated that the breathing exercises had helped them to come to terms with remote education (and the associated strains) with 12% reporting that the Breathing Breaks had helped them “a lot”. 18% of the children supported their home practice by showing the breathing exercises to family members and friends^15^. Thus, a sizeable number of children transferred the Breathing Breaks to their homes and established a somewhat regular practice which may have strengthened their neural pathways^46^. Future studies are needed to confirm that practice time mediates the results of MBIs on arithmetic.

Children’s arithmetic test scores correlated moderately to strongly with their grades in mathematics, especially at T2 and T3. At T1 the correlation was weaker because some teachers had known the children for only a short time. Contrary to our expectations, teacher ratings in mathematics did not improve in the IG any more than in the ACG, possibly because they are subjective assessments which contain more systematic errors (i.e., biases) than standardized tests. In addition, the 5-point rating scale might not have been sensitive enough to capture interindividual differences in children’s achievements in math. Whereas teacher ratings are a summative assessment, the arithmetic test LVD-M 2–4 is a curriculum-based test which is likely to measure students’ progress (and interindividual differences between them) with higher sensitivity^54^. On the downside, the LVD-M 2–4 was limited to measuring children’s arithmetic skills whereas teacher assessments encompassed their mathematics performance overall. The effect of the BBI is therefore limited to children’s achievements in arithmetic.

The present study confirmed the PISA report that parents’ educational attainment impacts on their children’s mathematics performance^31^. Lower mathematics performance in children with parents with low educational attainment may be due to poorer access to educational materials and less financial resources for tutoring. Social disparities increased during the pandemic because children depended on having access to a computer and on private teaching materials during distance schooling^1^. Because T-values were used for each grade level, controlling for age or grade was not necessary.

Strengths of the current study include decomposing the somewhat fuzzy concept of mindfulness into a set of 15 exercises on breathing and body awareness, which were easy to lead for teachers and fun to perform for children. Further strengths involve using both an objective test (of arithmetic) and teacher ratings to assess students’ performance over time. Methodological strengths include realizing a RCT, accounting for the nested structure of the data which controls for classroom effects, as well as using an active control group which counteracted unspecific treatment effects. That intervention effects on children’s performance in arithmetic were obtained even though ACG children colored mandalas^55^, speaks for the strength of the BBI, because coloring mandalas may also stimulate self-regulation. Furthermore, the effects of the teacher-led Breathing Breaks could be demonstrated at the level of the children and at the level of the classroom over a longer period of time.

Limitations include the sample of children from one region of Germany, and the use of the arithmetic test of the previous grade at T1 which decreased comparability with T2 and T3. Generalizability and internal validity were limited by school closures between T2 and T3 in which Breathing Breaks could not be continued in the classrooms. Because all classrooms in each school were assigned to the same treatment condition, school-related influences cannot be ruled out. Because teachers were aware of whether their class was in the IG or the ACG, teacher expectancy effects cannot be excluded. The large variances around the group means suggest that additional variables need to be considered, which may moderate the results, such as math teachers’ familiarity with the class (which may have influenced their ratings), students’ prior experiences with breathing exercises, or students’ appreciation of the Breathing Breaks. Future studies with larger and more varied samples are needed to replicate the present results under normal circumstances, to study dose–response relations with practice in school and at home, and to include possible moderators.

The school based BBI emerged as a promising remedial approach to address the decline of children’s mathematical competencies in Germany^1^ which is easily accessible for teachers and efficient in terms of time and costs. Future studies should investigate the mechanisms underlying the effects of the BBI on children’s math performance i.e., the effects of the different mediators (EFs, stress, math anxiety, self-regulation, academic self-concept, prosocial behavior) alone and in combination on children’s performance in arithmetic and other areas of mathematics.

## Data Availability

Data will be made available upon reasonable request by contacting katharina.voltmer@leuphana.de.
